# Supplementary material for: Are carbon emissions trading and green financial instruments synergistic? -Comprehensive quantitative research based on content analysis
Source: PLoS One. 2024 Mar 7;19(3):e0298601. doi: 10.1371/journal.pone.0298601 (PMC10919601; doi:10.1371/journal.pone.0298601)
Supplement: S1 Data — (ZIP) [file pone.0298601.s001.zip › Data/地级市Z_F工作报告.zip!/║■─╧/│╗╓▌╩╨/└┤╫╘╣1⁄2╓┌║┼í╛┬φ┐╦╩2╛▌═°í┐_2010.docx]

各位代表：

现在，我代表市人民政府向大会报告工作,请予审议,并请市政协各位委员和其他列席人员提出意见。

一、2009年工作回顾

2009年，是郴州“两城”建设的开局之年，也是我市经济发展最为困难、富有成效的一年。一年来，面对国际金融危机的严峻挑战，在市委的坚强领导下，全市人民深入学习实践科学发展观，坚定信心、迎难而上，抢抓机遇、化危为机，实现了经济社会大发展，步入了科学跨越的快车道。

（一）积极应对，化危为机，综合实力快速提升。面对2008年底以来严峻复杂的经济形势，迅速实施“千亿投资计划”、“百亿城建计划”，组织开展交通、城市、产业建设“三大会战”，投资拉动经济增长12.8个百分点。面对工业企业生产经营困难，开展“企业服务年”等活动，出台有色金属储备、设立工业发展基金等扶持政策，引导企业“抱团取暖”，协调银企合作，工业拉动经济增长8.8个百分点。面对居民消费需求不足，出台促进消费的一揽子政策措施，消费拉动经济增长7.2个百分点。面对上半年出口贸易大幅下降，加快发展加工贸易，加工贸易进出口总额逆势增长88.7%。面对返乡农民工骤增、市内企业歇工待岗等形成的就业压力，采取“以创业带就业、以内需扩就业、以社保稳就业、以培训促就业”等措施，实现了城乡就业稳定增长。在应对国际金融危机的重大考验中，我们以坚定的信心、超常的举措，创造性地贯彻落实中央、省委、省政府和市委的决策部署，见事早、判断准、行动快，率先实现了经济形势回升向好，多项主要经济指标增幅创历史新高，综合经济实力迈上新台阶。全市生产总值突破800亿元、达821.5亿元，同比增长16%，是改革开放以来郴州经济增长最快的一年，增幅居全省第一位；工业增加值379.4亿元，增长20.2%；财政总收入突破80亿元、达80.2亿元，一般预算收入48.8亿元，分别增长22%和27.1%；资兴、北湖、桂阳、永兴、苏仙等5个县市区生产总值超过百亿元、财政总收入超过7亿元。

（二）扩大投资，狠抓项目，发展后劲明显增强。把加大投资作为扩内需、保增长、强基础、惠民生的重中之重，开展“项目建设年”活动，完成全社会固定资产投资597亿元，同口径增长99.5%，增幅居全省第一位。以“5加2”、“白加黑”、“晴加雨”的精神和毅力，实施投资2000万元以上（房地产投资5000万元以上）的主要建设项目530个，完成年度投资399.3亿元。武广高铁和嘉新、永安、汝广、嘉宁等6条干线公路顺利通车，厦蓉、衡武、宜凤等高速公路建设快速推进。中心城区武广新区一期、郴资桂大道改扩建一段、城市规划展览馆等25个城市基础设施项目竣工或基本完工。华磊光电、华新水泥、金贵银业、郴西500千伏变电站等104个工业技改和产业升级项目、34个农业产业化项目、37个现代服务业项目竣工投产或完成年度投资计划。储备重大前期项目112个，总投资1808亿元。我们建成了一批规划多年而没有建成的项目，干成了一批多年想干而没有干成的大事！

（三）先行先试，改革创新，发展机制更具活力。争取省委、省政府出台了支持郴州承接产业转移先行先试“34条”政策并抓紧落实，新引进转移型企业214户。强力推进标准厂房建设，建成面积167.6万平方米，在建面积117.4万平方米，当年出租率达62.5%，新增就业岗位2万多个。完善开放平台，郴州海关开关，郴州公路口岸开放，郴州出口加工区叠加保税物流功能并启动二期建设，检验检疫监管职能延伸，供港澳农产品验放场加快建设，郴州成为全省乃至中西部开放平台最全、功能最优的市州之一。成功举办东莞招商会、广州政策解读会、中国（郴州）有色金属产业发展高峰论坛等重要招商活动，开展“集中招商月”活动，华信有色、太平洋建设、上海杉杉、深圳中邦等战略投资者落户郴州。实际利用外资4.65亿美元，到位内资156.2亿元，分别增长19.2%和13.9%。加大改革创新力度。深化投融资体制改革，搭建融资平台44个，创新20多种新型金融业务和BT、BOT等多种融资模式，新增融资168亿元，增长98.2%；全面推进中心城区城管行政执法和环卫管理体制改革，形成了市、区两级政府共抓城市管理的合力；创新园区管理体制，市有色产业园区（郴州出口加工区）托管苏仙区白露塘镇；深化农村各项改革，流转土地332.4万亩；市属国有企业基本完成改革改制任务；增值税转型、燃油税、交通体制和城区财政体制等各项改革进展顺利。先行先试为郴州科学跨越发展注入了新的活力和动力。

（四）调整结构，转型产业，发展质量显著提升。三次产业结构由上年的14.6∶49.1∶36.3调整为13.3∶50.1∶36.6。工业发展提速增效。新增年产值过亿元的工业企业78户，其中华润电力超过50亿元；有色、电子等六大优势产业增加值增长24.5%，规模工业增加值增长24.1%、利润增长25.8%；新增高新技术企业4户，高新技术产业增加值增长23.6%。我市被列为国家知识产权试点城市。永兴县被认定为国家稀贵金属再生利用产业化基地。强力推进矿产资源整合，煤矿整顿关闭兼并重组取得重大阶段性成果。加快节能减排重点工程建设，8个县城污水处理厂和市污水处理厂扩建工程全部投入运行，城乡垃圾无害化处理设施建设全面启动，列入湘江流域综合整治的109个项目和198家企业基本整治到位。资兴市被列入全国资源枯竭型城市转型试点。森林覆盖率62.75%，提高0.25个百分点。万元生产总值能耗下降6.5%，规模工业能耗下降15.5%，化学需氧量排放量下降2.8%，二氧化硫提前一年完成“十一五”减排任务。服务业繁荣发展。社会消费品零售总额349.9亿元，增长19.3%。公路水运货物周转量增长11.5%，房地产投资、销售面积分别增长40.9%、48.7%，游客接待人数、旅游综合收入分别增长27.9%、34.9%；金融机构新增贷款增长102.3%，保险费收入增长9.9%。建成标准化农家店800个，家电下乡实现销售收入2.2亿元。现代农业加快发展。粮食总产量188.5万吨，主要农产品供应充足。新增市级以上农业龙头企业39户、省级农民专业合作示范社10户，农民专业合作组织发展到1380个，市级以上农业龙头企业总产值和利润分别增长40.1%、62.9%。补贴推广农机具2.6万台。非公有制经济加快发展，增加值、实缴税金分别增长20.1%和34.3%

（五）扩容提质，统筹发展，城乡面貌变化喜人。突出中心城区建设，统筹城乡发展，掀起了新一轮城镇建设热潮。在全省率先完成城市总体规划（2009-2030年）、土地利用总体规划（2006-2020年）修编，为建设100平方公里、100万人口规模的区域中心城市奠定了坚实基础。中心城区以城东新区和武广新区为重点，以郴资桂大道改扩建、相山大道、市体育中心、环城森林生态景观工程等重点项目建设为标志，实施基础设施项目77个，改造背街小巷20条，完成投资34.7亿元，是我市城建史上投资规模最大、建设速度最快的一年。中心城区全面启动“三创”工作，开展“城市管理规范年”活动，整治交通秩序、市容环境，拆除违法建筑19.4万平方米，跻身全省卫生城市甲类行列。县城和小城镇加速扩容提质。加快推进新农村建设。完成新农村建设规划200个，新增市级新农村建设示范村22个、省级2个，100%的乡镇和87.2%的行政村通水泥（油）路；完成人工造林50万亩、病险水库除险加固68座，新解决18.8万农村人口安全饮水问题，新建沼气池1.03万口；培训农民104万人次；减少贫困人口5.1万人。

（六）改善民生，成果共享，社会大局和谐稳定。市政府确定的33项为民办实事项目全部完成或超额完成任务。城镇居民人均可支配收入13727元，农民人均纯收入4519元，分别增长9.9%和10.2%。全面启动国家创业型城市试点工作，新增城镇就业5.6万人，稳定农村劳动力转移就业91万人，城镇登记失业率3.92%。完善社会保障体系，落实企业退休人员养老保险和城乡低保待遇提标，开展农民工和新型农村养老保险试点，改扩建乡镇敬老院38所、农村“五保之家”10所，城镇居民基本医疗参保率、“新农合”参合率分别提高到81%、93.5%。新建（筹集）廉租房104.4万平方米、经济适用房17.1万平方米，解决了2.1万户城镇低收入家庭住房困难；实施灾民住房重建和农村危房改造3910户，中心城区裕后街棚户区改造、东江库区移民避险搬迁、采煤沉陷区综合治理、农房统保等民生工程顺利推进。加快教育强市建设，中心城区新增义务教育学位8000个，创建义务教育合格学校103所，改扩建中等职业学校5所，郴州职业技术学院顺利搬迁，高中阶段入学率突破85%，资助家庭经济困难学生3.8万人。推进医药卫生体制改革，实施9项基本公共卫生和6项重大公共卫生服务项目，新改扩建乡镇卫生院50所、村卫生室35个，社区卫生服务体系进一步健全，市第一人民医院改扩建、县级医院标准化建设加快推进，甲型H1N1流感疫情得到有效控制。市文化中心、市博物馆、市图书馆开馆，建成乡镇综合文化站54个、农家书屋319个。人口计生工作跻身全省先进行列，人口自然增长率6.27‰。广播电视覆盖率96%以上。我市运动员在全运会上再夺金牌，打破三项世界纪录。价格监管和服务取得新成效。切实加强安全生产，实施煤矿全面停产整顿，安全生产形势总体平稳。集中整治信访突出问题，推进“法治郴州”创建，完善社会治安防控体系，加强应急管理，打击“黑恶势力”、“两抢一盗”等各类犯罪，城区“两抢”发案率下降58.4%，全市维稳工作由全省第10名上升到第3名。民族宗教、外事侨务、审计、统计、人防、档案、气象、地震、水文、移民、地方志和妇女、儿童、老龄人、残疾人、慈善等事业都取得了新的成绩。驻郴部队和民兵预备役部队为地方经济社会发展做出了新的贡献。

（七）开放决策，绩效考核，开放型政府建设成效明显。出台《郴州市人民政府科学民主决策程序暂行规定》，邀请市人大代表和市政协委员列席、市民代表旁听市政府重要会议，建立健全市政府领导接听“市长公开电话”、接待信访群众、接收网民邮件并督办落实的工作机制和政府新闻发言人制度，依法对城市规划、项目建设等重大事项进行公示、听证。认真执行市人大及其常委会的决议决定，自觉接受其监督；健全市政府与市政协和各民主党派、工商联、无党派人士的联络机制，主动接受民主监督。办理市人大代表建议245件、市政协委员提案242件，办复率100%。密切联系工会、共青团、妇联、科协等群众团体。全面启动以市政务服务中心为平台、以“两集中、两到位”为重点的行政审批制度改革，推进网上审批和电子监察系统建设，市级行政许可事项减少61项，审批时限缩减50%以上。加强廉政建设和公务员队伍管理，开展公开承诺、民主评议活动，实施绩效考核，政务环境不断优化。大力整治项目建设施工环境，投诉率下降61.9%。社会公众对市直单位机关作风平均满意率81.6%，比2007年提高15个百分点。

各位代表，过去的一年，我们面临的困难和挑战超过预期，取得的成绩好于预期。这些成绩的取得，凝聚了全市人民的智慧和汗水，得益于各级各方面的关心和支持。在此，我代表市人民政府，向全市各族人民，向驻郴解放军、武警部队官兵，向所有关心、支持和参与郴州建设发展的海内外朋友，表示衷心的感谢并致以崇高的敬意！

回顾一年来的工作，我们深深体会到：越是在困难的时候，越要坚持科学跨越发展不动摇，敢于自我加压，确立科学跨越目标，坚定率先突破、率先发展信心，增创发展新优势。越是在困难的时候，越要坚持改革开放不停步，勇于先行先试，创新体制机制，承接产业转移，集聚创新资源，在重点领域和关键环节取得新突破。越是在困难的时候，越要坚持狠抓项目建设不松劲，强力拉动经济快速增长，为长远发展增强后劲。越是在困难的时候，越要坚持惠及民生不偏向，始终团结和依靠人民群众，全力办好群众急切盼望的大事实事，让群众共享发展成果，形成攻坚克难的强大合力。越是在困难的时候，越要坚持建设开放型政府不懈怠，完善政府决策机制，创新政府工作流程，大兴求真务实之风，全面提升政府创造力、执行力、驱动力和公信力，以实干造福于民，以实绩取信于民。

我们也清醒地看到，前进中还存在不少困难和问题：民间投资不活跃，居民消费后劲不足，经济增长内生动力有待增强；调结构、转方式任务繁重，现代服务业发展滞后，园区经济规模偏小，矿业整合、环境保护、节能减排压力增大；经济运行生产要素偏紧，财税减收增支因素增多，统筹区域发展的调控措施有限；民生保障处于较低水平，城乡居民增收难度大，部分企业经营和群众生活较为困难；安全监管存在薄弱环节，社会治安形势仍然严峻，经济发展环境有待进一步优化；政府的服务质量和办事效率与群众的期望还有较大差距。对此，我们将采取有效措施认真加以解决。

二、2010年工作任务

2010年，是完成“十一五”规划、为“十二五”规划启动实施奠定良好基础的关键之年，也是推进“两城”建设大干三年的攻坚之年。当前，一方面，我们要看到“后危机时代”经济形势的复杂性，世界经济复苏的基础尚不稳固，我国经济增长的内生动力仍显不足，我市调结构、转方式、扩内需、惠民生的压力增大。另一方面，国家继续实施积极的财政政策和适度宽松的货币政策，加大对中部地区的支持；省委、省政府要求郴州争当湘粤赣省际区域发展“领头羊”，进一步加大对郴州的支持力度；我市项目建设如火如荼，发展势头强劲有力，干部群众干事创业的激情迸发。特别是武广高铁的通车，标志着郴州跨入了“高铁时代”，必将在珠三角与长株潭两大经济体加速对接、两大城市群加速融合、走廊产业经济带加速形成中，迎来快速发展、科学跨越的全新时代。只要我们抢抓机遇、科学谋划、加压奋进，在发展中促转变，在转变中谋发展，就一定能够在新的起点上实现新的跨越。

今年政府工作的总体要求是：以邓小平理论和“三个代表”重要思想为指导，深入贯彻落实科学发展观，围绕“两城”建设目标，深入推进“一化三基”，放手先行先试，提质量、增效益，调结构、转方式，优投资、扩消费，惠民生、促和谐，力争“湘南率先、省际领先、湖南争先”，实现经济社会又好又快发展，推进科学跨越、富民强市，争当湘粤赣省际区域发展“领头羊”。主要预期目标是：地区生产总值增长15%；财政总收入和一般预算收入分别增长15%；全社会固定资产投资增长35%；社会消费品零售总额增长20%；外贸进出口总额增长21%；城镇居民人均可支配收入和农民人均纯收入分别增长9%、7%；城镇登记失业率控制在4.5%以内；居民消费价格涨幅控制在3%左右；人口自然增长率控制在7‰以内；完成“十一五”节能减排目标。

（一）强力推进项目建设，保持投资稳定较快增长。继续开展“项目建设年”活动，深入推进交通、城市、产业建设“三大会战”，全面完成“千亿投资计划”。全年安排总投资2000万元以上（房地产投资5000万元以上）的主要建设投资项目816个，年度计划投资612.3亿元，其中总投资过亿元项目357个，年度计划投资474.6亿元。交通建设，安排项目39个，年度计划投资115亿元。重点推进衡茶吉铁路和厦蓉、衡武、宜凤、岳汝等4条高速公路，以及桂临、宜嘉、资五等9条干线公路建设，适时启动桂道、三南2条高速公路和107国道宜章绕城线建设。基本完成通村公路改造。抓紧做好郴州机场项目前期工作。通过加快交通基础设施建设，形成交通新优势，链接“高铁新干线”。城乡建设，安排项目217个，年度计划投资156.1亿元。中心城区年内完成郴资桂大道改扩建二段和三段、相山大道、青年大道和国庆路等提质改造、五岭广场人防工程等项目建设，加快实施武广新区二期、卜里坪路、107国道城区段等道路建设和提质改造，启动东江二期引水工程。通过加快城市基础设施建设，构建区域中心城市，融入“高铁城市圈”。产业建设，安排项目438个，年度计划投资284.4亿元。重点推进高斯贝尔扩建、华磊光电LED、骏峰LCD、天沅化工等高新技术产业和工业技改项目，衡郴燃气管线、中石化储油库、城网改造、邓家塘和城北220千伏变电站等能源项目，舜华鸭业扩建、新湘农养殖、南方资源农业科技园等农业产业化项目，湘南国际物流园、宝山矿山公园、农贸市场提质改造、电子商务等现代服务业项目，以产业园区和产业转型对接“高铁经济带”。民生事业，安排项目122个，年度计划投资56.8亿元，比上年增加68个项目、28.1亿元投资。年内完成市体育中心、市演艺中心、市广电演播厅等项目建设，加快市第一人民医院改扩建、市中医院搬迁、县级医院标准化建设、市会展中心、市科学馆等项目建设，新改扩建乡镇计生服务所39所。新建3G通信基站354个，建设气象灾害预警信息发布接收系统136个、电子显示屏100块。加大项目融资和监管力度。完善和规范融资平台，继续运用BT、BOT、信托等融资方式，进一步鼓励和引导民间资本参与基础设施、产业项目和生态环境建设。通过发行企业债券、扩大金融机构贷款、吸引民间资金、资本市场融资、争取国家和省级预算资金等，力争年内融资200亿元。适度增加政府偿债准备金。完善重点项目推进协调机制，加强项目全程质量、安全和工程监理，确保工程建设进度和质量。坚决控制产能过剩行业投资。加强对政府投资项目的审计监管。结合“十二五”规划编制，超前谋划、储备、开发一批重大项目。优化项目建设环境。深入开展“优化重点项目建设环境年”活动，进一步优化政务环境，严肃查处行政机关及其工作人员在项目建设中的违纪违法行为；进一步优化施工环境，健全项目建设施工环境责任制和工作机制；进一步落实重点项目建设治安环境责任制，依法惩治强揽工程、强买强卖、恶意索要、阻工闹事等违法犯罪行为。规范项目征地拆迁行为，完善征地拆迁补偿安置政策。

（二）大力开展先行先试，不断激发发展活力。进一步落实先行先试“34条”政策，在更大范围拓展和深化先行先试政策，力争在调整经济结构与转变发展方式、扩大内需与促进外需、破解要素瓶颈与激发内生动力、加强政府调控与完善市场机制等方面取得重大突破，为全省创造新经验。加快开放承接产业转移。加强承接载体建设，市财政继续安排5000万元专项资金，引导金融信贷和民间资本，新建标准厂房180万平方米，其中政府主导投资建设标准厂房100万平方米，在9个重点园区集中布局、形成规模、加强配套。加快郴州出口加工区二期、供港澳农产品验放场等平台建设。开通铁海联运，完善口岸功能，提高大通关效率。大力发展加工贸易，力争加工贸易总额比上年翻两番。扩大外贸出口总量，优化进出口产品结构，增加高新技术和高附加值产品出口。加大力度招商引资。有针对性地选择招商引资方式，发挥协会、商会和驻外机构的招商作用。举办东莞招商会、上海招商会、“异地商会或高端台商郴州行”等重大招商活动，继续开展“集中招商月”活动，加强与央企、省企的对接，着力引进一批矿产品精深加工、电子信息、加工贸易、机械制造、基础设施等领域的重大项目和龙头企业，引进一批区域性总部企业或研发、销售中心。完善重大项目全程代办和绿色通道制度，加快项目落地。严格落实招商引资考核制度和奖励政策，力争实际利用外资5亿美元、到位内资170亿元。深化改革创新体制机制。切实抓好国有资产分类监管和市属国有企业改革改制扫尾工作。扶持非公有制经济加快发展，进一步放宽市场准入，从招投标、政府采购等方面保障非公企业的平等参与权，依法保护民间资本的合法权益。完善中小企业信用担保体系，引导社会资金参与组建商业性中小企业担保机构，鼓励非公企业组建互助式股份制担保机构。推进郴电国际在资本市场定向增发，支持符合条件的企业加快上市。积极做好财政省直管县配套改革。全面实施中心城区财税新体制。加快公务消费改革。推进义务教育学校、公共卫生、基层医疗卫生事业单位绩效工资改革。进一步理顺市区行政管理体制，加大管理重心下移，有序推进城市规划、建设、管理和国土资源、教育等体制改革。创新园区管理体制，赋予市有色产业园区（郴州出口加工区）、市经开区市级综合经济管理权限。全面实施城镇居住证制度，鼓励有稳定职业和收入的农民工及其子女转为城镇户口，纳入城镇社会公共服务体系。推进农村土地流转等各项改革，基本完成集体林权制度改革。稳妥推进水、电、成品油等资源性产品价格和环保收费改革。全面完成政府机构改革，积极推进事业单位改革。

（三）突出产业结构调整升级，切实转变经济发展方式。开展“新型工业化奋进年”活动，加快实施“千百十工程”， 进一步加大对企业的帮扶力度，力争工业增加值增长20%、规模工业总产值突破千亿元。壮大提升优势产业。推动矿产资源向战略投资和精深加工集中，重点支持郴州氟化学10万吨萤石及6万吨无水氟化氢、桂阳银星10万吨铅冶炼、华信有色10万吨铅锌冶炼等矿业精深加工项目建设，加快稀土开发；继续推进铋、白银等行业整合重组，引导组建钨、钼集团；支持永兴国家稀贵金属再生利用产业化基地建设，加快筹建矿产地储备中心和有色金属交易中心、锌期货交割库，举办中国（郴州）有色金属交易博览会、中美中小企业发展论坛，打造“中国有色金属之都”。积极引进工程机械、装备制造龙头企业或配套企业，支持郴州粮机、富士电梯等企业和嘉禾机械装备制造基地建设发展。加快改造提升锻铸造、建材、食品等传统产业。大力培育战略性新兴产业。把电子信息、新材料、新能源、节能环保等作为战略性产业来抓。加快电子信息产业发展，引进龙头企业，加强产业配套，重点培育光伏、集成电路、芯片制造、传感网等产业集群。大力发展新材料产业，依托矿产资源优势，引进和培育新材料大项目、大企业，重点支持湘香锡业、杉杉新材料、金箭焊料等项目建设。推进新能源产业发展，加快仰天湖风力发电、凯迪生物质能等项目建设，合理开发小水电，抓好郴州核电项目前期工作。积极发展环保装备制造业、资源综合利用产业、洁净产品制造业和环境服务产业。推动生物医药产业发展。实施“三四一二”科技创新工程，加强科技创新平台建设，支持有条件的企业实施重大科技专项、产学研专项和重点科技项目，全面推进国家知识产权试点城市建设。力争高新技术产品增加值增长30%以上。做大做强园区经济。坚持优化布局、错位发展、产业集聚、市县联动，推进园区资源整合，加快园区基础设施、标准厂房及配套设施建设，大力招商引资，培育优势产业，提高园区产业集聚度。综合运用规划、财政、信贷等调控手段，引导新上产业项目向省级产业园区布局、生产要素向园区流动、优惠政策和产业引导资金向园区倾斜、审批权限向园区下放，促进园区经济加速发展。力争两年内园区工业总量占全市规模工业的40%以上。加快发展现代服务业。大力发展现代物流业，推进电子信息、矿产品、建材、农产品等生产性物流园区发展，引进和培育大型商贸流通企业。积极发展现代金融业，力促交行、中信、光大、浦发、广发、长沙银行等在郴设立分支机构，加快组建农村商业银行、小额贷款公司，扩大村镇银行发展规模，规范发展证券、保险业，新增贷款增长20%。着力发展现代商务、信息服务、文化创意和会展业。积极发展低碳经济。在全社会大力倡导和鼓励低碳生产、低碳消费模式。加快节能减排重点工程建设，深入开展企业节能行动，加快城镇污水处理厂及管网配套、城乡生活垃圾无害化处理设施建设，确保化学需氧量削减6.14%。深入推进矿产资源整合，突出抓好煤矿整合重组，继续抓好柿竹园、玛瑙山、小垣、黄沙坪、三十六湾等矿区综合治理，治理病险尾矿库156座。整治关停小水泥、小冶炼等高耗高排企业。完成湘江流域郴州段水污染治理三年行动计划，实施重金属污染专项治理。加大城乡环境保护力度，加快森林生态恢复建设，增加森林碳汇，完成造林50万亩。加强国家级主体功能区建设，建立生态补偿机制，加强东江湖水资源及周边环境保护。大力发展循环经济，抓好重点园区、企业循环经济试点，支持资兴国家可持续发展示范区、永兴国家循环经济示范园建设。

（四）加大城镇建设力度，提升城镇发展水平。严格实施城乡规划。加强城市总体规划宣传，提高投资者和市民的知晓率。组织实施城市总体规划特别是近期建设规划，完成中心城区给排水、城市夜景、道路交通等专项规划，加快编制城市园林绿地、环卫、公交等专项规划。加强控制性规划编制工作，重点完成东河组团、观山洞片区、苏仙区工业项目区等控规编制，中心城区控规覆盖率90%以上。加强各县市区的乡（镇）、村规划编制工作，完成县乡土地利用总体规划修编。规划一经批准即具有法律效力，任何单位和个人不得擅自更改。加强中心城区建设管理。拟安排城市基础设施项目135个，年度计划投资94亿元，提前完成“百亿城建计划”。采取政府主导、市区共建、社会参与的方式，重点建设改造62条城市主次干道、12条背街小巷、33座垃圾站、47座公厕和地下管网、截污干管、交通管理设施、“城中村”、河道治理等项目，实施城区山头绿化、“三湖一园”等生态建设工程。围绕“三创”目标，深入开展“城市管理规范年”活动，全面加强城市精细化管理，推进街道管理网络化、社区管理物业化、城管工作数字化。继续开展交通秩序、市容秩序、违章建筑、户外广告、汽车尾气排放等综合整治。广泛开办“市民讲坛”，培育提升市民文明素质。通过加强城市建设和管理，实现市容环境水平明显提升、城市功能品质明显提升、人居环境质量明显提升。加快推进郴资桂城镇群和小城镇发展。重点实施郴资桂城际快速公路提质改造工程，实行统一规划、分段共建、整体推进，助推郴资桂经济一体化。以规划为引导、项目建设为支撑、产业发展为重点、制度创新为保障，推进郴资桂区域交通、通讯、金融、环保等基础设施融合，加速资兴、桂阳融城步伐，培育200万人口规模的城镇群，构建市域经济核心增长极，打造湘粤赣省际区域综合竞争力最强的城镇群。加快县城和中心城镇扩容提质，实施城镇环境综合治理工程，每个县市区建设1个示范性小城镇。

（五）坚持统筹城乡发展，加快建设现代农业。加强农业综合生产能力建设。改革农村土地管理制度，稳步推进土地综合整治。加强农村道路、电网、水利、高标准基本农田等基础设施建设。实施92座病险水库除险加固工程，全面完成大中型和重点小I型水库除险加固任务。加大农业科技投入和科技创新力度，深入推进“十百千”农村科技示范工程，实施农田测土配方施肥工程500万亩。完善农产品质量安全监测监管体系，加快防灾减灾体系建设。做好防汛抗旱、森林防火、动植物防疫、农产品质量安全监管、农机推广、涉农保险等工作。加快转变农业发展方式。加强耕地保护和粮食生产能力建设，确保粮食总产量200万吨以上。加大农业结构调整力度，大力发展农产品精深加工业，推进农业产业化经营，重点建设10大农业龙头企业、10大优势农业产业、20个农产品基地和20个特色农业品牌。积极引导农产品加工企业向农产品加工园区集聚，形成规模效应。拓宽农产品销售渠道，重点培育30户农民专业合作示范社，每个县市区组建1个以上农产品物流公司。着力发展现代烟草农业。积极发展生态农业、园艺农业、休闲农业和外向型农业，每个县市区建设1个以上高标准的现代农庄。努力增加农民收入。全面落实强农惠农政策，扩大涉农补贴规模，促进农民收入稳定增长。加大农村劳动力就业信贷和财税支持，培育农民收入新的增长点，培训农民80万人次。加大扶贫开发力度，改善老少边穷地区和库区居民生产生活条件，增强自主发展能力，减少贫困和低收入人口5万人以上。积极推进新农村建设。加强乡村规划编制工作，推进旧村庄、“空心村”改造，整治农村违规建房，引导农村人口向小城镇和中心村适度集聚。解决16万农村人口安全饮水问题，新建沼气池1万口。实施“金太阳”示范工程，为边远山区3000户“无电户”安装2万千瓦太阳能供电装置。调动社会力量参与新农村建设，扩大村级公益事业财政奖补试点范围，重点建设好600个新农村示范村和11个示范区，突出抓好郴资桂城际快速公路新农村示范区建设。

（六）扩大居民消费需求，增强经济发展内生动力。提高居民消费能力。通过落实提高城乡居民收入政策、促进产业发展、发展劳务经济、增加就业岗位、健全职工工资增长机制，多渠道增加居民特别是中低收入居民的收入。加大对低收入家庭的补贴和救助力度。加强价格调控和价格监管，保持粮食、生猪等主要农副产品的合理价格水平。培育扩大消费热点。抢抓郴州融入珠三角和长株潭“1小时城市圈”的机遇，谋划新的消费增长点。稳定和扩大住房消费，加快保障性住房建设，鼓励开发中低价位、中小套型普通商品房，适度发展中高档精品楼盘，支持居民自住和改善性住房消费。大力发展旅游文化产业，开展“旅游项目建设攻坚年”活动，建设100个、总投资200亿元的旅游项目，承办好2010年中国（湖南）红色旅游文化节，加速与珠三角、长株潭和东南亚等旅游线路的无缝对接，打造国内知名休闲度假目的地。合理引导和促进汽车消费。积极推进服务性消费，加快发展现代物流、服务外包、电子商务、会展经济等现代服务消费，扩大教育培训、医疗保健、文化娱乐、体育健身、家政服务等新兴消费，提升餐饮、住宿等传统消费。有效引导“产地消费”，提高本地产品市场份额。大力促进农村消费，继续做好家电、汽摩下乡工作，落实家电、汽车以旧换新政策，增加农机购置补贴，开展“工业品下乡、农产品进城”促销活动，促进农村消费升级。改善消费环境。加强市场监管，规范市场秩序，维护消费者合法权益。推进社会信用体系建设，优化金融生态和信用消费环境。加强市场规划和建设，中心城区加快建设现代化、高品位的大型综合购物中心和专业市场，满足不同消费群体的需求；今明两年全面完成中心城区农贸市场规划和新建改造提质，彻底改变“脏乱差”面貌。继续实施“万村千乡”市场工程和新农村流通服务网络工程，新建标准化农家店700家、覆盖90%以上的行政村，力争三年内村邮站覆盖率80%。

（七）更加注重保障和改善民生，促进社会和谐稳定。坚持不懈地为民办实事，重点在社保、扶贫、移民、医疗、饮水、环保、法律援助等方面实施一批民生工程，让群众共享发展成果。积极扩大就业。落实创业扶持政策，降低创业门槛，促进全民创业，以创业带动就业。加强城乡就业公共服务体系建设，加大对下岗失业人员、就业困难人员、农民工的职业技能培训和就业服务。落实大学生就业创业扶持政策，统筹做好转业退伍军人、城镇新增劳动力和农村转移劳动力的就业指导。大力开发公益性岗位，完善就业援助制度，重点帮扶零就业家庭、残疾人、低保对象、下岗失业人员和就业困难大学生。新增城镇就业5.2万人、农村劳动力转移就业7.6万人。提高社会保障水平。企业退休人员基本养老金人均月新增120元，中心城区城乡居民低保补助人均月分别提标20元、10元。建立健全最低生活保障标准与物价涨幅联动机制。开展新型农村养老保险和社区居家养老服务试点，扶持发展养老服务事业，三年内实现乡乡有敬老院目标。积极发展社会福利和慈善事业。扩大城镇职工、居民医保覆盖面，所有原国有关闭破产企业退休人员和困难企业职工全部纳入城镇职工基本医疗保险，所有在校大学生纳入城镇居民医保范围。城镇居民医保和“新农合”财政补助标准提高到每人120元，“新农合”参合率达到92%、实际住院补偿率50%。加强住房保障体系建设，新建（筹集）廉租房71.8万平方米、经济适用房8万平方米以上，启动农村土坯房改造试点，做好农房统保工作。加快旧城区和国有工矿棚户区改造、东江库区移民避险搬迁、采煤沉陷区治理，改造棚户房21.7万平方米，全面完成中心城区裕后街棚户区改造。大力推进教育强市建设。坚持教育优先发展战略，健全义务教育经费保障机制，促进各级各类教育协调发展。突出抓好城区中小学扩容、中小学校舍安全、合格学校建设三大工程，建设义务教育合格学校100所，力争中心城区新增义务教育学位1万个。完善县级职教中心，加快郴州职教实训基地建设，支持郴州职业技术学院、技师学院加快发展。加强教师队伍建设。完善教育资助体系，确保每个家庭经济困难学生不因贫困辍学。加快医药卫生事业发展。落实医药卫生体制改革近期重点实施方案和五项重点任务。建立健全卫生经费保障机制，完善疾病防控、医疗救治、公共卫生突发事件应急和卫生执法监督等公共卫生体系。开展免费婚前医学检查。加强乡镇卫生院和社区卫生服务中心标准化建设。推进农村药品“两网”建设。强化食品药品监管执法。扶持中医药事业发展。有效防控艾滋病、结核病、甲型H1N1流感等传染病。加强医护人员培训和医德医风建设。统筹发展各项事业。实施文化强市战略，推进“文化惠民工程”，加快公共文化服务体系建设，大力发展文化事业和文化产业。新建乡镇综合文化站52个、农家书屋655家。完善人口计生工作长效机制，推进基层计生服务体系建设，统筹解决人口问题。广泛开展全民健身运动，承办第七届全省少数民族运动会。组织编制“十二五”规划。开展第六次人口普查。积极创建全国残疾人工作示范城市。重视和加强民族宗教、外事侨务、审计、统计、文物、档案、气象、地震、水文、科普、地方志和妇女、儿童、老龄人等工作。加强国防动员、国防教育、人民防空、拥军优抚安置和国家安全工作，支持驻郴部队建设。全力维护社会稳定。严格落实安全生产责任制，狠抓安全隐患排查整治，严厉打击非法违法生产行为，有效防范和坚决杜绝重特大事故发生。更加注重和改进信访工作，拓宽和畅通社情民意表达渠道，着力解决信访突出问题。深入开展法制宣传教育，创建“法治郴州”。推进平安郴州建设，强化社会治安综合治理，推动工作重点向基层下移，继续向重点部位和企业派驻维稳工作组；加快市、县两级电子监控系统联网，完善突发事件应急管理和治安防控机制，依法打击“黑恶势力”、“两抢一盗”等各种犯罪，维护社会稳定。

（八）深入推进开放型政府建设，提高人民群众满意度。着力提高开放式决策水平。完善政府决策体系，落实科学民主决策机制，实行重大决策专家咨询论证、社会公示和听证、合法性审查、集体决定及后评价制度，完善市人大代表和市政协委员列席、市民代表旁听市政府重要会议制度。认真执行人大及其常委会的决议决定，自觉接受人大及其常委会监督和政协民主监督，依法向人大及其常委会报告工作，及时向政协通报情况。提高人大代表议案、建议和政协委员提案办理质量。主动听取各民主党派、工商联、无党派人士和群众团体的意见建议。接受新闻舆论监督和社会监督，支持监察、审计部门依法监督。深入推进政府信息公开，提高政府工作透明度。着力提高公共服务水平。加快转变政府职能，创新政府管理方式，深入推进以“两集中、两到位”为重点的行政审批制度改革，进一步提高行政效率。稳步推进政府采购、建设工程交易、水利工程交易等中心进驻市政务服务中心，构建全市统一的公共资源交易中心。加快建设覆盖全市的电子政务网，年内市、县两级网上审批和电子监察系统建成并实现联网。依法规范行政裁量权，建立健全依法行政考核机制。加大公共财政对民生领域和社会事业的保障力度。着力提高政府绩效水平。切实加强公务员队伍建设。各级政府和全体政府工作人员要更加注重执行、更加注重实干、更加注重实绩，一以贯之、一抓到底、抓出成效。加强行政效能建设，加大政务督查和行政问责力度，确保政令畅通和工作落实。切实加强党风政风廉政建设，严肃查处违纪违法案件，坚决纠正损害群众利益的不正之风。带头厉行节约、勤俭办事，严格控制一般性开支，坚决反对和制止铺张浪费。我们的政府是人民的政府。我们一定要殚精竭虑、勤奋务实，努力建设让全市人民满意和支持的开放型政府。

各位代表，关于把郴州建设成为“湖南最开放城市、湘粤赣省际区域中心城市”的《议案》已提请大会审议决定，“两城”建设《战略规划纲要》（征求意见稿）已印发各代表团讨论。本次会议审议后，我们将进一步修改完善《战略规划纲要》，并按法定程序提请市人大常委会审议决定。

各位代表，站在新的起点上面向未来，郴州人民更加豪情满怀、意气风发。让我们在中共郴州市委的正确领导下，团结和依靠全市人民，坚定信心，加压奋进，扎实工作，奋勇拼搏，朝着“两城”建设的宏伟目标阔步前进，以更优异的成绩向郴州建市15周年献礼！
